# Holding dysregulation in mind: How maternal mind‐mindedness relates to regulatory symptoms and disorders in infancy

**DOI:** 10.1002/imhj.70020

**Published:** 2025-05-18

**Authors:** Anna Katharina Georg, Lea Alexandra Schlömp, Monika Forck, Miriam Binder, Svenja Taubner

**Affiliations:** ^1^ Clinical Psychology and Psychotherapy for Children and Adolescents University of Tübingen Tübingen Germany; ^2^ Institute for Psychosocial Prevention Heidelberg University Hospital Heidelberg Germany; ^3^ Institute of Psychology Heidelberg University Heidelberg Germany

**Keywords:** infant regulatory disorders, maternal mind‐mindedness, mentalizing, parenting stress, psychological distress, الاضطرابات التنظيمية لدى الرضع ، الوعي الذهني الأمومي، ضغوط التربية، الاضطراب النفسي، التركيز الذهني, 婴儿调节障碍, 母亲心理心智化, 育儿压力, 心理困扰, 心智化, Troubles de la régulation chez l'enfant, mentalité maternelle, stress parental, détresse psychologique, mentalisation, Regulationsstörungen im Säuglingsalter, Mütterliche Mind‐Mindedness, Elterliche Belastung, Psychische Belastung, Mentalisierung, 乳児の調整障害、母親のマインド・マインデッドネス、育児ストレス、心理的苦痛、メンタライジング, trastornos regulatorios infantiles, atención mental materna, estrés de crianza, angustia sicológica, mentalización

## Abstract

This study examined the role of maternal mind‐mindedness (MM) – the tendency to ascribe mental states to one's child – in infant regulatory symptoms and disorders and the moderating role of parenting stress and global psychological distress. A better understanding of these relationships may inform prevention and intervention programs. The interactional MM measure (appropriate and positive mind‐related comments) was applied in a clinical group with regulatory disorders (ClinGrp; *N* = 124) and a healthy comparison group (CompGrp; *N* = 31) with mothers and their infants aged 4 to 15 months in Germany. Group differences between the ClinGrp and the CompGrp were calculated. Positive MM was higher in the CompGrp than in the ClinGrp. Appropriate and positive MM were negatively related to infant regulatory symptoms. Psychological distress was negatively linked to appropriate and positive MM. Parenting stress and psychological distress did not moderate the relationship between appropriate MM and regulatory symptoms. Lower‐quality MM characterizes parent–infant interactions in the context of regulatory symptoms and disorders. Implications for future research on the valence of mind‐minded comments, longitudinal studies on the protective role of MM, possible child effects, and clinical implications are discussed.

## ملخص

تناولت هذه الدراسة في دور الوعي الذهني الأمومي ‐(MM) الميل إلى إدراك الحالة الذهنية للطفل وفهم أفكار ومشاعر الطفل‐ في ظهور الأعراض والاضطرابات التنظيمية لدى الرضع والدور الوسيط لضغوط التربية والاضطرابات النفسية العامة. قد يؤدي الفهم الأفضل لهذه العلاقات إلى إثراء برامج الوقاية والتدخل. تم تطبيق مقياس الوعي الذهني الأمومي (MM) التفاعلي (تعليقات مناسبة وإيجابية متعلقة بالحالة العقلية) على مجموعة تجريبية تعاني من اضطرابات تنظيمية (العدد = 124) ومجموعة مقارنة صحية (العدد = 31) من الأمهات وأطفالهن الرضع الذين تتراوح أعمارهم بين أربعة أشهر و15 شهراً في ألمانيا. تم حساب الفروق في المجموعة بين المجموعة التجريبية والمجموعة الضابطة. كان معدل الوعي الذهني الإيجابي أعلى في المجموعة الضابطة من المجموعة التجريبية. وارتبط الوعي الذهني الأمومي المناسب والإيجابي ارتباطاً سلبياً بالأعراض التنظيمية للرضع. وارتبطت الضائقة النفسية ارتباطاً سلبياً بالوعي الذهني الأمومي المناسب والإيجابي . ولم يتوسط متغير الإجهاد الوالدي والضيق النفسي في العلاقة بين الوعي الذهني الأمومي المناسب والإيجابي والأعراض التنظيمية. تُميِّز جودة منخفضة من الوعي الذهني الأمومي التفاعلات بين الوالدين والرضيع في سياق الأعراض والاضطرابات التنظيمية. تناقش الدراسة التطبيقات العملية للنتائج والبحوث المستقبلية حول تكافؤ التعليقات الذهنية والدراسات الطولية حول الدور الوقائي للتفاعل بين الوالدين والرضيع، والآثار المحتملة على الطفل، والتطبيقات الاكلينيكية.

## 摘 要

本研究探讨了母亲心理心智化 (MM) ——即母亲将心理状态归因于其孩子的倾向——在婴儿调节症状与障碍中的作用, 以及育儿压力和整体心理困扰的调节效应。深入理解这些关系有助于为预防和干预项目提供依据。研究在德国开展, 采用互动性MM测量 (适当且积极的与心理相关的评论) , 对象包括一个调节障碍临床组 (ClinGrp;N = 124) 和一个健康对照组 (CompGrp;N = 31) , 母婴对象的婴儿年龄为4至15个月。研究计算了ClinGrp与CompGrp之间的组间差异。结果显示, CompGrp中的积极MM水平高于ClinGrp。适当且积极的MM与婴儿调节症状呈负相关。育儿压力和心理困扰并未调节适当MM与调节症状之间的关系。在调节症状与障碍背景下, 较低质量的MM是亲子互动的特征之一。研究还讨论了关于心理心智化评论效价的未来研究方向、MM保护作用的纵向研究、可能的儿童效应以及临床意义。

## Résumé

Cette étude s'est penchée sur le rôle de l'Orientation Mentale (OM) Maternelle – la tendance à attribuer des états mentaux à son enfant – dans les symptômes et troubles de la régulation du nourrisson, ainsi que le rôle modérateur du stress parental et de la détresse psychologique globale. Une meilleure compréhension de ces relations pourrait éclairer les programmes de prévention et d'intervention. La mesure de OM interactionnelle (commentaires appropriés et positifs liés à l'esprit) a été appliquée dans un groupe clinique ayant des troubles de régulation (ClinGrp; N = 124) et un groupe de comparaison sain (CompGrp; N = 31) chez des mères et leurs nourrissons âgés de quatre à 15 mois en Allemagne. Les différences de groupe entre le ClinGrp et le CompGrp ont été calculées. Un OM positive et appropriée s'est avérée être plus élevée dans le CompGrp que dans le ClinGrp. Les OM appropriées et positives se sont avérées négativement liées aux symptômes de la régulation du nourrisson. La détresse psychologique était négativement liée à une OM appropriée et positive. Le stress parental et la détresse psychologique n'ont pas atténué le lien entre une OM appropriée et les symptômes de régulation. Une OM de qualité inférieure caractérise les interactions parent‐enfant dans le contexte des symptômes et des troubles de la régulation. Les implications pour les recherches futures sur la valence des commentaires de l'orientation mentale, les études longitudinales sur le rôle protecteur de l'OM, les effets possibles chez les enfants et les implications cliniques sont discutées.

## Zusammenfassung

Diese Studie untersuchte die Rolle der mütterlichen Mind‐Mindedness (MM) ‐ der Tendenz, dem eigenen Kind mentale Zustände zuzuschreiben ‐ bei Regulationssymptomen und ‐störungen bei Säuglingen sowie die moderierende Rolle von elterlicher Belastung und globaler psychischer Belastung. Ein besseres Verständnis dieser Zusammenhänge kann eine bessere Grundlage für Präventions‐ und Interventionsprogramme liefern. Die interaktionelle MM‐Messung (angemessene und positive mentale Kommentare) wurde in einer klinischen Gruppe mit Regulationsstörungen (ClinGrp; N = 124) und einer gesunden Vergleichsgruppe (CompGrp; N = 31) mit Müttern und ihren Säuglingen im Alter von vier bis 15 Monaten in Deutschland angewendet. Es wurden Gruppenunterschiede zwischen der klinischen und der Vergleichsgruppe berechnet. Positive MM war in der Vergleichsgruppe höher als in der klinischen. Angemessene und positive MM standen in einem negativen Zusammenhang mit Regulationssymptomen des Kindes. Psychische Belastung stand in einem negativem Zusammenhang mit angemessener und positiver MM. Elterliche Belastung und psychische Belastung beeinflussten den Zusammenhang zwischen angemessener MM und Regulationssymptomen nicht. Eine geringere Qualität der MM kennzeichnet Eltern‐Kind‐Interaktionen im Zusammenhang mit Regulationssymptomen und ‐störungen. Es werden Implikationen für die zukünftige Forschung über die Valenzen von mentalen Kommentaren, Längsschnittstudien über die schützende Rolle von MM, sowie mögliche Auswirkungen auf das Kind und klinische Implikationen diskutiert.

## 要旨

この研究では、母親のマインド・マインデッドネス (MM) —　わが子に心の状態を帰属させる傾向 — が乳児の調整症状や調整障害にどのように関係しているか、さらに育児ストレスや全般的な心理的苦痛を和らげる役割について検討した。これらの関係性をより深く理解することは、予防や介入プログラムに役立つ可能性がある。相互作用的MMの測定 (適切かつ肯定的な心に関するコメントについて) を、ドイツにおいて、調整障害を持つ臨床群 (ClinGrp; N = 124) と健康な比較群 (CompGrp; N = 31) の母親とその4〜15か月の乳児に対して適用した。ClinGrpとCompGrpの群間差を算出したところ、CompGrpの方がClinGrpよりも肯定的なMMが高いことがわかった。適切かつ肯定的なMMは、乳児の調整症状と負の相関があった。心理的苦痛は適切かつ肯定的なMMの低下と関連していた。育児ストレスや心理的苦痛は、適切なMMと調整症状との関係に影響を与えなかった。調整症状や調整障害の文脈での親子相互作用においては、MMの質の低さが特徴的であった。今後の研究について、MMコメントの価値に関する研究や、MMの保護的役割に関する縦断研究、子どもへの影響の可能性、および臨床的意義が検討されている。

## Resumen

Este estudio examinó el papel de la atención mental prestada por la madre (MM) – la tendencia a adscribirle estados mentales al propio hijo – en síntomas y trastornos regulatorios infantiles y el papel moderador del estrés de crianza y la angustia sicológica global. Una mejor comprensión de estas relaciones pudiera servir de información para programas de prevención e intervención. La medida interactiva MM (apropiados y positivos comentarios relacionados con la mente) se aplicó en un grupo clínico con trastornos regulatorios (ClinGrp: N = 124) y a un grupo comparativo saludable (CompGrp: N = 31) con madres y sus infantes de edad de cuatro a 15 meses en Alemania. Se calcularon las diferencias de grupo entre el ClinGrp y el CompGrp. Una positiva MM fue mayor en el CompGrp que en el ClinGrp. La apropiada y positiva MM fue relacionada negativamente con los síntomas regulatorios infantiles. La angustia sicológica fue negativamente ligada a la apropiada y positiva MM. El estrés de crianza y la angustia sicológica no sirvieron de moderadores de la relación entre la apropiada MM y los síntomas regulatorios. Una más baja calidad de MM caracteriza las interacciones progenitor‐infante en el contexto de síntomas y trastornos regulatorios. Se discuten las implicaciones para la futura investigación sobre la base de la valencia de comentarios acerca de la atención mental prestada, los estudios longitudinales sobre la base del papel de protección de MM, los posibles efectos en el niño, así como las implicaciones clínicas.

## 1 INTRODUCTION

Infant regulatory symptoms and disorders such as persistent excessive crying or sleeping, feeding, and sensory processing disorders place considerable strain on the parent–child dyad and are associated with future mental health problems in children (e.g., Skovgaard et al., 2007; Winsper et al., 2020). Furthermore, research demonstrates a link between infant regulatory symptoms and parents’ experience of parenting stress as well as psychological distress (e.g., Georg, Schröder‐Pfeifer et al., 2021), with parenting stress, in turn, showing a robust link to dysfunctional parenting behavior (e.g., Mak et al., 2020). A better understanding of which parenting factors are associated with infant regulatory symptoms and play a role in differentiating clinical groups with regulatory disorders from healthy comparison groups helps to identify targets for prevention and intervention programs.

Parents’ capacity to represent and interpret their infants’ mental states could facilitate more effective regulatory behavior in their children. In the case of infant regulatory symptoms and disorders, however, it can be challenging for parents to understand and appreciate their infants’ needs and affect expressions. Research of the last two decades increasingly paid attention to a specific feature of the parent–infant interaction that describes these processes: Mind‐mindedness (MM) can be seen as the parents’ spontaneous proclivity to acknowledge their child in terms of mental states such as thoughts, feelings, or desires (Meins, 1999). MM is entailed in the concept of parental mentalizing, which is defined as the parents’ capacity to treat the child as a psychological agent (Sharp & Fonagy, 2008). From a child's perspective, having a mind‐minded parent means that their mental states and needs will more likely be recognized, mirrored in language, and acted upon (Bigelow et al., 2015). The use of more mind‐minded descriptions as well as more appropriate mind‐minded comments have been shown to stimulate healthy social development in children (e.g., Meins et al., 2013) and contribute to more sensitive parenting (Farrow & Blissett, 2014) and to infant secure attachment development (Zeegers et al., 2017). In contrast, the use of more non‐attuned mind‐minded comments could be a risk factor for the development of emotional and behavioral problems in children (e.g., Colonnesi et al., 2019).

**Key findings**

- This study provides initial evidence that the quality of maternal utterances about a child's mental states during interactions is related to infant regulatory symptoms as well as infant regulatory disorders.
- Appropriate and positive MM were negatively related to infant regulatory symptoms. Positive MM was observed more often in mother–child interactions in infants without regulatory disorders (vs. with regulatory disorders). The group comparison needs replication using a larger control sample.
- More parent psychological distress was negatively associated with appropriate and positive MM during mother–child interactions, possibly pointing to functional MM being susceptible to higher levels of psychological distress.

**Statement of relevance**

This study explores the concept of mind‐mindedness (MM) and its association with infant regulatory symptoms and disorders to inform parent–infant interventions aimed at improving infant mental health. Parent–infant interactions with infants, both with and without regulatory disorders, were coded for MM. The results suggest that less appropriate and less positive MM characterizes the interactions of mothers with infants with more regulatory symptoms. Practitioners working with affected families may focus on reinforcing appropriate and positive MM, although further research is needed.

### 1.1 Mind‐mindedness

MM describes the tendency of a parent to see the child as an individual with their own mind, which is evident in the parent's communication with and representations of the child (Meins & Fernyhough, 2015). There are two approaches to measure MM: an interactional and an interview approach. In the interview, parents are asked to describe their child. The proportion of references to the child's mental states in the narrative is then used as an indicator for MM (mind‐minded descriptions; Meins et al., 1998). The interactional measure, in contrast, assesses parents’ spontaneous verbal references to the infant's mental states during a free‐play interaction with their infant (mind‐minded comments; Meins et al., 2001). The interactional measure allows evaluating the accuracy of mind‐minded comments according to the raters’ perception of the parent–child interaction (McMahon & Bernier, 2017). Mind‐minded comments are appropriate if the comments are considered compatible with the infants’ behavior (e.g., the mother comments, “You like the doll” while the child is playing happily with a doll). Contrarily, non‐attuned MM manifests in comments that reflect a misperception of the child's mental states (e.g., the mother comments, “You like the doll” while the child is not paying attention to the doll but, instead, explores another toy). Using a modified version of the MM measure, Demers et al. (2010a, 2010b) proposed coding the valence of mind‐minded comments (positive, negative, or neutral) in the interview and interactional measure (e.g., “stubborn,” “moody,” or “temperamental” is classified as a negative mind‐minded comment and “sensitive” or “joyful” as positive). In the interactional approach, the context and the mother's tone of voice are also taken into account (Demers et al., 2010b).

### 1.2 Mind‐mindedness, child self‐regulation, and child emotional and behavioral problems

Various explanations regarding the importance of acknowledging the child's mental states for the development of the child's self‐regulation and mental health have been put forward. For example, it has been proposed that appropriate MM helps parents to regulate their child's negative affect and behavior by acting as a verbal scaffold, thereby supporting the development of self‐regulation (McMahon & Bernier, 2017). MM can also be regarded as a type of parental mirroring in language. According to the affect mirroring theory, maternal mirroring behavior is thought to facilitate infants’ understanding of their own emotions and support their self‐regulation (Gergely & Watson, 1996, 1999). Thus, appropriate MM may reflect parents’ successful interpretation of their child's affect and may help them regulate their child and, thereby, contribute to the development of the child's self‐regulation. Child self‐regulation, in turn, relates to concurrent and subsequent mental health outcomes (Robson et al., 2020). Furthermore, a direct relation between MM and child emotional and behavioral difficulties has been proposed. It was argued that parents who are more mind‐minded, may experience difficult child behavior as less irritating and might have more resources to intervene and redirect potential conflictual interactions with the child (McMahon & Bernier, 2017).

Based on these considerations, several cross‐sectional studies examined the association between the different MM indicators and children's emotional and behavioral problems, supporting the view that using more appropriate and positive MM and less negative MM is related to fewer emotional and behavioral problems in children. Camisasca et al. (2018) found that higher appropriate MM was linked to less internalizing and externalizing behaviors in 17‐month‐olds. A community group of mothers with children aged 3 to 5 years used more MM descriptions and less negative MM compared to mothers of children with emotional and behavioral problems (Walker et al., 2012). Beyond preschool years, Hughes et al. (2017) reported that the use of more (positive) mind‐related descriptors was associated with less disruptive behavior in 12‐year‐old children. This effect was moderated by family adversity, showing a more pronounced negative association between MM and children's emotional and behavioral problems in the context of high versus low family adversity.

Longitudinal studies also investigated the protective effect of appropriate MM on children's mental health development with slightly varying results. Meins et al. (2013) found that higher appropriate MM at 8 months predicted both fewer externalizing and internalizing problems at 44 months in families with low socio‐economic status. Colonnesi et al. (2019) found that more appropriate MM at 30 months predicted less externalizing but not internalizing problems at 4.5 years in a community sample of mothers and fathers.

### 1.3 Mind‐mindedness and parental distress

According to the dual‐system theory of information processing, mentalizing is more automatic in stressful situations, resulting in non‐reflective and biased assumptions about the self and others (Luyten et al., 2020). Increased general psychological distress and parenting stress can cause parents to be distracted and emotionally aroused during parent–child interactions, thereby probably giving way to more automatic versus controlled mentalizing. Regarding MM, this suggests that parents show less appropriate and more non‐attuned MM under higher distress.

Studies on the relation between MM and psychological distress often focused on depression, with a meta‐analysis revealing no evidence for a significant association between depressive symptoms and MM (interview and observational measure) in general but specifically for depressed versus nondepressed samples (Georg, Meyerhöfer, et al., 2023). The authors concluded that compromised MM might occur in the clinical spectrum of depression. One study focusing on general psychological distress found no association with MM descriptions (Barreto et al., 2016). Schacht et al. (2017) investigated MM in mothers hospitalized for a range of severe mental illnesses, who produced less appropriate and, in another sample, additionally more non‐attuned MM compared to healthy mothers.

Evidence on the association between parenting stress – defined as the parental experience of being overburdened (Tröster, 2011) – and MM is also inconclusive. Some studies reported moderate to strong negative correlations between parenting stress and mind‐related descriptors of the child (e.g., McMahon & Meins, 2012) and appropriate mind‐related comments (Camisasca et al., 2017), while other studies reported non‐significant results (Larkin et al., 2021; Suttora et al., 2020). Associations with valence indicators of MM are similarly indefinite. More parenting stress was related to more negative MM and less positive MM (e.g., Demers et al., 2010a; Larkin et al., 2021). In contrast, Kirk and Sharma (2017) did not find a significant relation between positive MM and parenting stress. Overall, studies show a tendency for an association between parental stress (i.e., psychological distress and parenting stress) and MM; however, more research is necessary to figure out under which circumstances this association is present.

A few cross‐sectional studies investigated whether parental distress moderated the association between appropriate MM and children's emotional and behavioral problems. In trauma‐exposed mothers who did not meet the full criteria for post‐traumatic stress disorder (PTSD), higher appropriate MM was associated with fewer child emotional and behavioral problems than in mothers who met PTSD criteria (Easterbrooks et al., 2017). In a more recent study, Hobby et al. (2022) found that when mothers reported *high* parenting stress, increased appropriate MM was associated with fewer externalizing problems; in contrast, when mothers reported *low* parenting stress, increased appropriate MM was associated with fewer internalizing problems. Overall, these results indicate that parental distress seems to moderate the strength of the association between MM and child mental health outcomes: In some cases, the association was stronger under higher levels of parental distress; in some cases, it was stronger under lower levels of parental distress. More research is necessary to clarify which circumstances modulate the association between MM and child outcomes in which direction.

### 1.4 Mind‐mindedness and infant regulatory symptoms

Two studies examined MM in relation to child functioning and mother–child interactions in sleeping and feeding contexts, which is particularly relevant to the subject of infant regulatory symptoms and disorders investigated in the present study. One study found that more appropriate MM at 6 months predicted more reciprocal and less conflictual mealtime dynamics at 12 months (Yatziv et al., 2020). Furthermore, another study found that appropriate MM at 12 months was linked to children's sleeping consolidation at 3 and 4 years (Bordeleau et al., 2012). These results indicate that mind‐minded parents show more responsive caretaking behaviors in sleeping and feeding contexts.

However, the aforementioned studies investigated infants without mental health problems. Furthermore, to our knowledge, there are no studies on the associations between MM and regulatory symptoms and disorders in children below the age of 12 months. Also, studies rarely considered other parent variables, such as parental distress, as moderators regarding the relation between MM and child outcomes. This study aimed to address this gap by examining different indicators of MM and their association with children's regulatory symptoms and disorders and parental distress.

### 1.5 The present study

The present study extends previous research on the role of MM regarding infant mental health. We cross‐sectionally observed MM in mother–child interactions in a clinical group (ClinGrp) of mothers and their infants with diagnosed regulatory disorders and a comparison group (CompGrp) without the diagnosis of regulatory disorders to capture the range of infant regulatory symptoms from a more developmentally expectable lower end up to a clinically more severe level. At the same time, the group design allowed us to investigate possible categorical differences in parent–child dyads who experience regulatory disorders as opposed to healthy dyads. Given that elevated infant regulatory symptoms increase the risk of chronic parenting stress and psychological distress (e.g., Evers, Georg, et al., 2023; Georg, Schröder‐Pfeifer et al., 2021), we investigated their relationship with MM as well as the moderating role of parenting stress and psychological distress in this relationship.

In line with previous research (McMahon & Bernier, 2017), we expected rare instances of non‐attuned MM and thus focused on appropriate MM. Since the valence of MM may elucidate important qualitative differences in MM (Demers et al., 2010a), we planned to examine positive and negative MM. However, negative MM was observed rarely in our sample and, thus, was excluded from the analyses (similar to McMahon & Meins, 2012). This resulted in the following hypotheses:

1. In parents with healthy children, appropriate and positive MM will be higher than in parents of children with early regulatory disorders.
2a. Across both groups, more appropriate and more positive MM will be related to fewer infant regulatory symptoms.
2b. Across both groups, more parenting stress and more psychological distress will be related to less appropriate and less positive MM.

In line with Luyten et al.’s (2020) suggestion that mentalizing processes are affected by stress, and following previous moderation analyses focusing on appropriate MM (Easterbrooks et al., 2017; Hobby et al., 2022), we formed the following hypothesis:

3. Across both groups, the relationship between appropriate MM and regulatory symptoms is moderated by parenting stress and psychological distress.

## 2 METHOD

Our study has a cross‐sectional observational design. Data of the ClinGrp were derived from baseline assessments of a randomized‐controlled trial (RCT) testing the efficacy of a brief parent–infant intervention for treating early regulatory disorders (Georg, Cierpka et al., 2021). Families participated in the study between February 2014 and March 2018 at the Institute for Psychosocial Prevention at Heidelberg University Hospital. The data reported here were collected before the start of the intervention. The CompGrp, comprising parents with infants of the same age as the infants in the ClinGrp but without early regulatory disorders, were recruited between 2016 and 2017. The approval for this research was obtained from the ethics board of the Medical Faculty of Heidelberg University (No. S‐541/2013, approved November 4, 2013). This study was not preregistered.

The sample size of the ClinGrp was determined by a power analysis run for repeated‐measures analysis of variance for the interaction group (intervention vs. treatment as usual) by time (before vs. after intervention), which was done for the previous RCT (Georg, Cierpka et al., 2021). The sample size of the CompGrp was a priori set to *N* = 31 due to limited resources. The two sample sizes (ClinGrp, CompGrp) were therefore predetermined for different pragmatic reasons.

### 2.1 The sample

#### 2.1.1 Inclusion and exclusion criteria

Inclusion criteria for both groups required that the infant was four to 15 months old and was born full term (≥37 weeks of gestation). The pregnancy needed to be a singleton and primary caregivers had to speak German. Another inclusion criterion was the availability of a video recording of the free‐play session with sufficient quality to code MM. The quality criteria for the video recording were that (a) the mother spoke to her child in German, (b) the child did not predominantly sleep or be fed, (c) the mother and child were in front of the camera, and (d) the sound and image quality were sufficient.

Additional inclusion criteria for the ClinGrp were that the infants met diagnostic criteria for sleeping, feeding, or regulation disorders of sensory processing according to DC:0‐3 R (Zero to Three, 2005) or for a persistent excessive crying, sleeping, or feeding disorder according to the guidelines recommended by the German Society of Child and Adolescent Psychiatry, Psychosomatics and Psychotherapy (AWMF guidelines, 2007; AWMF No. 028/028). The diagnostic criteria were evaluated by trained clinicians based on a structured diagnostic interview that was developed for the RCT due to the lack of clinical interviews for infants at the time of the study. For more information on the interview, please see Georg, Cierpka et al. (2021). Participants of the ClinGrp were excluded when infants had a medical diagnosis that better explained the regulatory symptoms, a tentative diagnosis of fetal alcohol syndrome, or a diagnosed disability or developmental disorder. For the CompGrp, participants were excluded when infants met diagnostic criteria for regulatory disorders according to DC:0‐3 R or AWMF.

In both groups, the very high symptom severity of the mother (Symptom‐Check‐List‐90R‐S global severity index of *T* > 70) led to exclusion. See Georg, Cierpka et al. (2021) for a more detailed description and explanation of the inclusion and exclusion criteria of the ClinGrp.

#### 2.1.2 Recruitment

Families of the ClinGrp were referred from pediatric practices if parents reported significant crying, sleeping, or feeding difficulties. Additionally, some families self‐referred in response to public advertisement, websites, and flyers/posters distributed in gynecological, pediatric, and osteopathic practices, parent–infant groups, and crèches. A total of 190 primary caregivers underwent screening for eligibility via telephone. The primary caretakers were mothers in all cases. Mothers were informed about the study and were invited to participate if they were interested. Of these, 28 cancelled or did not show up. Eight families did not meet the inclusion criteria and were excluded. Video recordings were not available for five families, and in 25 families the quality of the video recordings was not sufficient to code MM. All participants provided informed consent before participating in the study. The final ClinGrp consisted of *N* = 124 mother–infant dyads.

Participants of the CompGrp were recruited via public advertisement, websites, and flyers/posters. A total of 31 mothers and one father attended the first assessment as primary caregivers and gave their informed consent. To obtain comparability with the ClinGrp, the father‐infant dyad was excluded from the current analyses. The final CompGrp consisted of *N* = 31 mother–infant dyads.

### 2.2 Procedure and assessments

Self‐report measures and behavioral diaries were mailed to mothers following the telephone screening. Clinical diagnostic assessments were performed by two psychologists in the ClinGrp and by a trained medical student (fourth author) in the CompGrp. The on‐site assessment included a clinical interview and a video recording of a standardized parent–infant interaction.

### 2.3 Instruments

#### 2.3.1 Maternal mind‐mindedness

Video‐taped mother–infant interaction sequences, each lasting 15 min, were coded using the Mind‐Mindedness Coding Manual (Version 2.2.; Meins & Fernyhough, 2015). Mothers received age‐appropriate toys and were instructed to play with their children as they normally would. Three raters (the fourth author and two medical/psychology graduate students), who were trained in the coding manual by E. Meins and subsequently underwent a reliability training outside of this study, independently coded the interaction sequences. Each utterance was classified as either mind‐related or not. According to the coding manual (Meins & Fernyhough, 2015), a comment is mind‐related if the mother talks explicitly about the child's mental life, emotion, and experience, or if she expresses possible ideas and thoughts of the child. Each mind‐related comment was classified as either appropriate or non‐attuned, depending on its evaluated consistency regarding the observed behavior of the child. Additionally, the valence of each mind‐related comment (irrespective of its accuracy) was considered and coded as either negative (e.g., “manipulative”), positive (e.g., “loving”), or neutral (e.g., “wilful”; Demers et al., 2010a, 2010b). In addition to the quality of the comment itself, the context, and the parent's tone of voice were taken into account (Demers et al., 2010b). In line with our hypotheses, we did not consider an indicator for neutral MM. Proportional scores were calculated to control for maternal verbosity by dividing the number of mind‐related comments by the total number of comments; thus, higher scores indicated more MM. Due to low valence‐specific proportional scores (*M* = .38, *SD* = .64 across both groups), positive MM scores were dichotomized (1 if present, 0 if not present). As negative MM was even less frequent (*M* = .02, *SD* = .11 across both groups) and present in only 5.69% of the interactions in the ClinGrp and none of the interactions in the CompGrp, negative MM was excluded from further analyses due to the low incidence.

To calculate the interrater reliability, 33 randomly selected video tapes from the ClinGrp were coded by all three raters. The raters were not blind to the group but to the hypotheses and other participant data; thus, we assume a low risk of bias. Inter‐rater agreement was examined using ICC for interval scaled data and Fleiss’ Kappa for dichotomized data (Gisev et al., 2013), leading to estimates for the total number of comments (i.e., maternal verbosity) of ICC = .93, number of mind‐minded comments of ICC = .97, appropriate MM of ICC = .96, non‐attuned MM of ICC = .96, and non‐dichotomized positive MM of ICC = .45. Thus, agreement was good to excellent except for non‐dichotomized positive MM where agreement was poor (Koo & Li, 2016). For the dichotomized positive MM variable that we used in our analysis, inter‐rater agreement was moderate with K = .45 (Landis & Koch, 1977).

#### 2.3.2 Infant regulatory symptoms

The Questionnaire for Crying, Feeding, and Sleeping (QCFS; Groß et al., 2013) assesses crying, sleeping, and feeding symptoms and parents’ dysfunctional co‐regulation behavior in response to the symptoms with 49 items, comprising three subscales. Higher scores indicate more regulatory symptoms, parental burden, and dysfunctional co‐regulation. Frequency questions regarding children's symptoms are rated on a four‐point Likert scale from 1 = *never*/*rarely* to 4 = *always*/*every day*. Parents’ perceived difficulties are rated from 1 = *not at all* to 4 = *a lot*. As we focused on children's symptoms, the subscale for fussing/crying and sleeping and the subscale for feeding were used. A mean score was calculated across both scales (total of 37 items), while the subscale for parental dysfunctional co‐regulation was not considered. Cronbach's α was good for the composite scale (.88).

#### 2.3.3 Psychological distress

The German Symptom Checklist (Symptom‐Checklist‐90R‐S; Franke, 2014) was used to assess self‐reported psychological distress, using the global severity index (SCL‐GSI). The SCL‐GSI indicates overall psychological distress within the last two weeks. The 90 items are rated on a five‐point Likert scale from 0 = *not at all* to 4 = *extremely*, and a mean score is calculated, with higher values indicating more psychological distress. Cronbach's α of the SCL‐GSI was excellent (.95).

#### 2.3.4 Parenting stress

The German Parenting Stress Index (PSI; Tröster, 2011) was used to assess parenting stress. The 48 items are rated on a five‐point Likert scale from 1 = *strongly disagree* to 5 = *strongly agree*. The questionnaire consists of two scales: a child domain and a parent domain. This study used the parent domain (PSI‐parent; mean score of 28 items), which taps into sources of stress related to parent characteristics. Cronbach's α was good (.88). Higher scores indicate more parenting stress.

### 2.4 Statistical analysis

R Studio and SPSS were used for all statistical analyses. The questionnaire data contained 1.7% missing values. Data imputation was performed assuming missingness at random, using multiple imputations by chained equations with 40 iterations (van Buuren & Groothuis‐Oudshoorn, 2011). Histograms were visually examined to check for the normal distribution of study variables. We used non‐parametrical tests for variables that were not distributed normally (SCL‐GSI, MM scores) and/or coded dichotomously (e.g., education, country of origin). We used *χ* ^2^‐tests, independent‐samples *t*‐tests, and Mann–Whitney *U*‐tests for group comparisons. All excluded dyads were part of the ClinGrp. Thus, to address potential systematic case exclusion, we compared the excluded dyads with the ClinGrp.

Mann–Whitney *U*‐tests and *χ* ^2^–tests were used to check for group differences regarding MM (Hypothesis 1). To test whether appropriate MM was associated with the QCFS (infant regulatory symptoms; Hypothesis 2a), PSI‐parent (parenting stress), and SCL‐GSI (psychological distress; Hypothesis 2b), Spearman's ρ correlation coefficients were calculated. Point‐biserial correlations were calculated for associations with the dichotomized variable positive MM. To test whether the relationship between appropriate MM and QCFS was moderated by PSI‐parent and SCL‐GSI (Hypothesis 3), assumptions of multiple linear regression (MLR) were checked first (Field, 2013). Linearity and independence of residuals were given. The variance inflation factor, which is an indicator of multicollinearity, was increased in the moderation models. However, this was expectable as the interaction term is a product of two of the other predictors (MM and PSI‐parent in Model 1 and MM and SCL‐GSI in Model 2). Based on McClelland et al. (2017) we considered the increased variance inflation factor as unproblematic. However, there were some outliers and small deviations from homoskedasticity, and normal probability plots indicated small deviations from the normality of the residuals. Therefore, we used the PROCESS macro version 4.3 for SPSS (Hayes, 2022) with percentile bootstrap confidence intervals (5,000 iterations) and a heteroscedasticity‐consistent standard error (HC4) as this method is more robust to violations of homoscedasticity and normality as well as outliers. We used the Johnson‐Neyman technique to estimate the interaction effects. Two MLRs were calculated using the QCFS score as the dependent variable. In Model 1, appropriate MM, PSI‐parent, and the interaction between appropriate MM and PSI‐parent were entered into the equation as predictors. We checked for correlations between the QCFS and appropriate MM and potential confounders (age and sex of child, number of siblings, marital status, age and education of mothers). Except for maternal age (*r* = –.24, *p* = .001) and maternal education (*ρ* = –.19, *p* = .011), correlations with the QCFS were near zero. Only maternal education was significantly associated with appropriate MM (*ρ* = .26, *p* < .001). Therefore, maternal age and education were included as control variables. Model 2 contained appropriate MM, SCL‐GSI, the interaction between appropriate MM and SCL‐GSI, and the control variables.

Research data cannot be made publicly available due to legal restrictions.

## 3 RESULTS

### 3.1 Study participants

Table 1 presents demographic characteristics, means and standard deviations of questionnaire scores, and results of the group comparisons. No significant group differences in demographic characteristics were found. The ClinGrp scored significantly higher on the QCFS, PSI‐parent, and SCL‐GSI (all *p*s < .001). In the ClinGrp, 28.2% (*n* = 35) of the infants met the criteria of persistent excessive crying, 97.6% (*n* = 121) showed a sleep onset or night waking disorder, 29.0% (*n* = 36) were diagnosed with a regulation disorder of sensory processing, and 23.4% (*n* = 29) met criteria for a feeding disorder. Of the infants, 33.9% were diagnosed with two and 18.2% with three or more regulatory disorders.

**TABLE 1:** Demographic and clinical characteristics in the clinical (*N* = 124) and comparison group (*N* = 31) and group comparisons.

Characteristic	ClinGrp	CompGrp	ClinGrp vs. CompGrp
Infant			
Female, *n* (*%*)	58 (46.77)	17 (54.84)	*χ* ^2^(1) = .36
Age, months, *M* (*SD*)	8.58 (3.16)	8.53 (3.01)	*U* = 1950
First‐born, *n* (*%*)	77 (62.60)	19 (61.29)	*χ* ^2^(1) = .00
QCFS, *M* (*SD*)	1.88 (.31)	1.37 (.16)	*t*(153) = –8.94***
Mother			
Age, years, *M* (*SD*)	33.26 (4.49)	34.90 (3.57)	*t*(153) = 1.89^+^
High school/higher education, *n* (*%*)	88 (70.97)	27 (87.10)	*χ* ^2^(1) = 2.58
German origin, *n* (*%*)	120 (96.77)	31 (100.00)	*χ* ^2^(1) = .14
Married, *n* (*%*)	96 (76.461)	25 (80.65)	*χ* ^2^(1) = .02
SCL‐GSI, *M* (*SD*)	.55 (.36)	.23 (.17)	*U* = 811***
PSI‐parent, *M* (*SD*)	2.93 (.64)	2.27 (.60)	*t*(153) = –5.88***

*Notes*: ClinGrp, Clinical Group; CompGrp, Comparison Group; QCFS, Questionnaire for Crying, Feeding, and Sleeping, subscale for crying, feeding, and sleeping; SCL‐GSI, Global Severity Index of the Symptom‐Check‐List‐90R‐S; PSI‐parent, parent domain of the Parenting Stress Index.

^+^ *p* < .10, *p < .05, **p < .01, and ***p < .001.

Comparisons between the ClinGrp and the participants excluded from the ClinGrp showed that the latter were more likely not to be of German origin, resulting in a final sample including only four non‐German mothers. This is because non‐German mothers mostly did not speak to their children in German during the video‐taped free‐play interaction, so we could not code their comments for MM (see 2.1.1 Inclusion and exclusion criteria). No significant differences were found for other variables (see supplementary material).

### 3.2 Mind‐mindedness and infant regulatory symptoms and disorders

Regarding Hypothesis 1, we found that positive MM was produced by significantly more mothers in the CompGrp than in the ClinGrp (*χ* ^2^(1) = 17.69, *p* < .001). In the ClinGrp, 28.2% of mothers showed positive MM compared to 71.0% in the CompGrp. No significant differences were found for appropriate MM. See Table 2 for descriptive statistics of MM indices per group and statistical indices of the Mann–Whitney *U*‐tests and *χ* ^2^‐tests.

**TABLE 2:** Descriptive statistics and comparisons of MM between the clinical and the comparison group.

	Clinical group (*N* = 124)				Comparison group (*N* = 31)					
	*M* (*SD*)	Median	IQR	Range	*M* (*SD*)	Median	IQR	Range	ClG vs. CG	*p*
MM	3.01 (2.19)	2.76	2.79	0–11.82	3.66 (2.05)	3.14	3.33	.78–8.42	*U* = 2282.5	.107
Appropriate MM	2.56 (1.89)	2.16	2.40	0–9.09	3.15 (1.99)	2.82		.47–7.90	*U* = 2266.5	.105
Positive MM	.28 (.56)	0	.33	0–2.93	.81 (.76)	.68	1.36	0–3.08	*χ* ^2^(1) = 17.69	**<.001**
Negative MM	.03 (.12)	0	0	0–.69	0 (0)	0	0	0		

*Note*: MM, mind‐mindedness. For group comparisons regarding MM and appropriate MM, Mann–Whitney *U* tests were calculated. For the group comparison of positive MM, a *χ* ^2^‐test was done on the dichotomized data. For negative MM, no group comparison was calculated as there was no negative MM in the comparison group. Bold letters indicate significance.

Regarding Hypothesis 2a, we found significant negative correlations of appropriate and positive MM, respectively, with regulatory symptoms (see Table 3).

**TABLE 3:** Correlations of study variables.

Variable	1.	2.	3.	4.	5.	6.
1. MM						
2. appropriate MM	.92^***^					
3. non‐attuned MM	.44^***^	.14^+^				
4. Positive MM	.19^*^	.19^*^	.06			
5. QCFS	–.18^*^	–.18^*^	–.08	–.16^*^		
6. PSI‐parent	–.10	–.14^+^	.02	–.07	.54^***^ ^,^ ^a^	
7. SCL‐GSI	–.20^*^	–.26^***^	–.01	–.18^*^	.54^***^	.64^**^

*Notes*: *N* = 153–155 due to missing values. Correlations of positive MM with other variables are point biserial as this variable was dichotomized. Due to skewed distributions, the remaining correlations were calculated by using Spearman's ρ except for ^a^ for which Pearson's *r* was calculated. MM, proportional mind‐mindedness; QCFS, Questionnaire for Crying, Feeding, and Sleeping, subscale for crying, feeding, and sleeping; PSI‐parent, parent domain of the Parenting Stress Index; SCL‐GSI, Global Severity Index of the Symptom‐Checklist‐90R‐Standard.

^+^ *p* < .10,

* *p* < .05,

** *p* < .01, and

*** *p* < .001.

### 3.3 Mind‐mindedness and parental distress

Regarding Hypothesis 2b, the PSI‐parent showed only a marginally significant negative correlation with appropriate MM and no significant correlation with positive MM. The SCL‐GSI was significantly negatively correlated with appropriate and positive MM (see Table 3).

### 3.4 Mind‐mindedness, parental distress, and regulatory symptoms

The results of the MLR analyses (Hypothesis 3) are displayed in Table 4. In Model 1, neither appropriate MM nor the interaction between the PSI‐parent and MM were significant predictors of the QCFS score. However, the PSI‐parent significantly predicted the QCFS score alongside with maternal age. A similar pattern emerged in Model 2. Appropriate MM and the interaction between the SCL‐GSI and MM did not significantly predict the QCFS score. However, the SCL‐GSI significantly predicted the QCFS over and above appropriate MM and maternal education, while the control variable maternal age was marginally significant.

**TABLE 4:** Results from MLR analyses to predict regulatory symptoms with appropriate MM, parenting stress (Model 1) and psychological distress (Model 2) as predictors.

Coefficients	Model 1	Model 2
(Intercept)	1.70^***^ (.27) [1.15; 2.24]	1.91^***^ (.29) [1.35; 2.47]
Appropriate MM	−.05 (.04) [−.13; .03]	−.02 (.02) [−.06; .02]
Mother's age	−.01^*^ (.01) [−.03; –.00]	−.01^+^ (.01) [−.02; .00]
Mother's education	−.02 (.05) [−.12; .09]	.05 (.06) [−.08; .17]
PSI‐parent	.23^***^ (.05) [.12; .32]	
Appropriate MM × PSI‐parent	.01 (.01) [−.02; .04]	
SCL‐GSI		.43^***^ (.13) [.18; .67]
Appropriate MM × SCL‐GSI		.03 (.05) [−.06; .13]
*R* ^2^ of the whole model	.32^***^	.27^***^
Δ*R* ^2^ of the interaction	.002	.001

*Notes*: *N* = 153. Unstandardized regression coefficients, (standard errors) and [95% confidence intervals]. SCL‐GSI, Global Severity Index of the Symptom‐Check‐List‐90R‐S; PSI‐parent, parent domain of the Parenting Stress Index.

^+^ *p* < .10,

* *p* < .05,

** *p* < .01, and

*** *p* < .001.

### 3.5 Exploratory analyses

**FIGURE 1:**
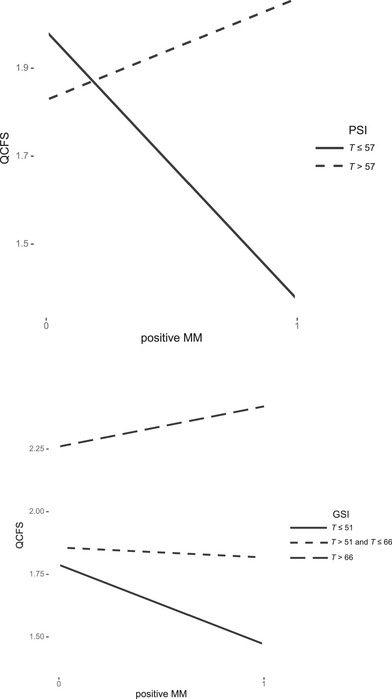
Interactions between positive MM and parenting stress / psychological distress to predict regulatory symptoms.

For exploratory analyses, we reperformed the MLR with positive MM as a predictor, given that positive MM was correlated with infant regulatory symptoms to a similar extent as appropriate MM. The results are displayed in Table 5. In Model 3 (Figure 1a), positive MM, the PSI‐parent, and the interaction positive MM × PSI‐parent significantly predicted the QCFS score. The Johnson‐Neyman approach revealed that the interaction was only significant for PSI‐parent ≤ 2.64 (*T* ≤ 57), indicating that the presence of positive MM was associated with lower QCFS scores only when mothers had lower levels on the PSI‐parent. A similar pattern emerged when considering the SCL‐GSI in Model 4 (Figure 1b). Positive MM, SCL‐GSI, and the interaction positive MM × SCL‐GSI significantly predicted the QCFS score. The interaction was significant for SCL‐GSI ≤ .31 (*T* ≤ 51) and SCL‐GSI > 1.18 (*T* > 66). This suggests that the presence of positive MM was negatively associated with QCFS scores only in the case of lower levels of SCL‐GSI, whereas it was positively associated with QCFS scores when mothers reported higher levels of SCL‐GSI.

**TABLE 5:** Results from MLR analyses to predict regulatory symptoms with positive MM, parenting stress (Model 3) and psychological distress (Model 4) as predictors.

Coefficients	Model 3	Model 4
(Intercept)	1.80^***^ (.26) [1.29; 2.31]	1.93^***^ (.26) [1.40; 2.45]
Positive MM	−.54^**^ (.18) [−.91; –.19]	−.24^**^ (.08) [−.39; –.09]
Mother's age	−.01^*^ (.01) [−.02; –.00]	−.01^+^ (.01) [−.02; .00]
Mother's education	−.05 (.05) [−.15; .05]	.03 (.06) [−.10; .15]
PSI‐parent	.19^***^ (.05) [.09; .27]	
Positive MM × PSI‐parent	.16^*^ (.07) [.04; .29]	
SCL‐GSI		.37^***^ (.09) [.19; .54]
Positive MM × SCL‐GSI		.45^**^ (.16) [.15; .78]
*R* ^2^ of the whole model	.34^***^	.31^***^
Δ*R* ^2^ of the interaction	.02^*^	.04^**^

*Notes*: *N* = 153. Unstandardized regression coefficients, (standard errors) and [95% confidence intervals]. SCL‐GSI, Global Severity Index of the Symptom‐Check‐List‐90R‐S; PSI‐parent, parent domain of the Parenting Stress Index.

^+^ *p* < .10,

* *p* < .05,

** *p* < .01, and

*** *p* < .001.

## 4 DISCUSSION

To our knowledge, this is the first study that investigated the role of MM in mother–infant interactions in relation to infant regulatory symptoms and disorders and the modulating role of parental distress. Past research has mainly focused on the association of MM with emotional and behavioral problems in older children (e.g., Walker et al., 2012) and neglected the particularly vulnerable population of infants with regulatory symptoms and disorders. Finding out whether MM is associated with infant regulatory symptoms and disorders and which circumstances possibly modulate this association could help to identify target variables for prevention and intervention programs in early childhood. Some but not all hypotheses were supported by our data.

### 4.1 Association between mind‐mindedness and infant regulatory symptoms

Regarding the relation between MM and infant regulatory symptoms, we found a pattern comparable to the results of the literature on child emotional and behavioral problems (e.g., Camisasca et al., 2018; Hobby et al., 2022): Higher appropriate MM and the presence of positive MM were associated with fewer regulatory symptoms. There are different possible explanations for this association. On the one hand, MM could exert an influence on infants’ regulatory capacity (parent effect). In longitudinal studies, appropriate MM predicted more positive feeding interactions (Yatziv et al., 2020) and sleep consolidation (Bordeleau et al., 2012) later on, indicating a protective effect of appropriate MM. Mothers who better recognize infants’ cues during free‐play interactions are probably more likely to adjust their behavior to the infant's needs and, thus, lower the risk for regulatory difficulties. Regarding valence‐specific indicators of MM, positive MM may reflect parents' capacity to positively frame challenging infant behavior, for example, by considering crying for long periods as alertness or sensitivity, thereby facilitating a warm and positive response (Demers et al., 2010a). This could positively affect children's self‐regulation (Bigelow et al., 2015) and protect against regulatory difficulties.

On the other hand, it is possible that regulatory symptoms and disorders negatively influence parental MM (child effect). Ambiguous infant signals or unpredictable responses to stimuli, which are more likely in regulatory disorders (Papoušek & von Hofacker, 1995), might increase parents' difficulties to accurately identify mental states in their infants, which could partly account for the relation between appropriate and positive MM and regulatory difficulties. In fact, a study with healthy mothers and infants revealed that behavioral markers of infants (i.e., gaze towards the mother) predicted increased child‐related mental state talk, a concept similar to mind‐related comments two months later (Northrup et al., 2019). Perceiving the child's mental states as less positive may also result from the child's irritable temperament. For appropriate MM, Meins et al. (2011) and Larkin et al. (2019) found no association with child temperament. Whether this is also the case for positive MM has not been investigated yet. More research is necessary to investigate appropriate and positive MM in relation to interindividual differences in children's behavioral signals and temperament evident during interactional sequences and longitudinally analyze potential child effects.

Importantly, however, the relationship between MM and regulatory symptoms may be bidirectional (cf. Northrup et al., 2019), containing both parent and child effects that reinforce each other over time. In the developing relationship with the child in the first year of life and beyond, parents' experiences with the child will probably impact their representation of the child (Larkin et al., 2021). Both parental factors (e.g., parental stress, own attachment experience) and child factors (e.g., postnatal adjustment difficulties, temperament) could play a role in these processes. Our cross‐sectional results indicate that functional facets of MM may be impaired in early parent–infant interactions in the context of infant regulatory symptoms and disorders. Future longitudinal studies may examine MM indicators, particularly the valence indicators, during early parent–infant interactions in infants at risk for developing regulatory disorders and investigate their role as a predictor for maladaptive child mental health trajectories in the context of parent and child variables.

### 4.2 Group differences in mind‐mindedness

Based on our result that parents with healthy children were more likely to produce positive MM than parents of children with regulatory disorders, a less positive parental view of the child's mental states may be particularly relevant to the clinical picture of regulatory disorders. It is surprising that we found no significant group differences regarding appropriate MM, even though the correlational analysis in the full sample revealed a small but significant association between appropriate MM and regulatory symptoms. This might suggest that, when considering the diagnosis of a regulatory disorder, positive MM plays a more important role than appropriate MM. However, the non‐significant group comparison could be a type‐II error due to limited variance and the relatively small size of the CompGrp. Therefore, a replication with a larger comparison group is needed.

In our sample, negative MM was not present in parents of healthy children and scarce in parents of children with regulatory disorders, with only 5.69% of the latter showing negative MM. Because of the very low frequency, we excluded negative MM from our hypotheses and analyses. On the one hand, it may seem encouraging that we found few negative mind‐related comments in a clinical sample of parents with regulatory disorders. On the other hand, based on the descriptive group differences in negative MM, there is limited evidence indicating that negative MM could be a specific risk factor for infant regulatory symptoms. The evidence for such a conclusion is scarce as other studies faced similar problems in detecting negative MM (e.g., Hobby et al., 2022) and excluded the variable from further analyses. Only two studies investigated the association between negative MM and child emotional and behavioral problems (Colonnesi et al., 2022; Konijn et al., 2020) and found no significant link. However, these studies focused on foster parents and used the interview measure; thus, comparability with our sample is limited. It is conceivable that negative MM is observed rarely in short free‐play interactions carried out in the laboratory due to social desirability. This might lead to an underestimation of its role in regulatory disorders and other child behavioral problems. Future studies should further investigate the valence ratings of MM and its relations to child mental health via observations under stress and in natural environments to potentially increase variability and reduce the impact of social desirability.

### 4.3 Associations between mind‐mindedness and parental distress

Mothers who reported more parenting stress showed marginally significantly less appropriate MM. These results add to the inconsistent literature on the relation between parenting stress and MM (e.g., Larkin et al., 2021; Kirk & Sharma, 2017). The marginally significant correlation could indicate that appropriateness is a stress‐vulnerable facet of MM, which could partly account for the link between parenting stress and dysfunctional parenting behavior (e.g., Mak et al., 2020). An alternative interpretation could be that more appropriate MM makes parents less prone to experiencing parenting stress. This was indicated by Hobby et al. (2022), who found that more appropriate MM at 10 months was associated with less parenting stress at 36 months. However, given the marginally significant correlation and the inconsistent literature, more research is needed on the link between parenting stress and appropriate MM.

We found no evidence in our study that more parenting stress is associated with the presence of positive MM. This result was unexpected as, for example, Larkin et al. (2021) found significant associations between parenting stress and positive MM. Future studies should consider these inconsistent findings and try to disentangle the direction of the tentative associations in the context of regulatory symptoms and disorders.

Regarding psychological distress, mothers who experienced more severe psychological symptoms were less engaged in appropriate and positive MM. This is in line with findings of lower appropriate MM and fewer mind‐minded descriptors in (clinically) depressed mothers (Georg, Meyerhöfer, et al., 2023). This could indicate that psychological distress inhibits functional MM. Psychological distress was also associated with less overall mind‐minded comments, irrespective of their accuracy; thus, the relationship between appropriate and positive MM might partly be explained by a reduction of overall MM.

The associations of parenting stress and psychological distress with MM indicators differed, with more robust results revealed for psychological distress. This could be due to parenting stress being more specific to demanding parenting contexts (Tröster, 2011) as opposed to psychological distress, which is a more general construct and less context‐specific (Franke, 2014); thus, the association between parenting stress and MM might be smaller than the association between psychological distress and MM in a relatively unchallenging free‐play parent–child interaction. This might be different in more stressful situations (e.g., during episodes of crying; cf. McMahon & Newey, 2018). Future research should compare how different aspects of MM relate to parental distress across varying levels of demands and stress, as well as in populations with differing degrees of parental distress (e.g., clinical samples of parents with mental health difficulties).

### 4.4 Moderation analyses

We neither found parenting stress nor psychological distress to moderate the association between appropriate MM and regulatory symptoms. While parenting stress and psychological distress predicted regulatory symptoms beyond the effect of appropriate MM, the interaction term with appropriate MM, as well as appropriate MM itself, were non‐significant predictors in the moderation analyses. The results could indicate that the association often found between regulatory symptoms and parental distress (Georg, Schröder‐Pfeifer et al., 2021) superimposes the negative relation between appropriate MM and infant regulatory symptoms; this should be investigated in future research. Our findings on the moderation effect diverged from previous studies involving older children (Easterbrooks et al., 2017; Hobby et al., 2022), which demonstrated that the association between appropriate MM and child emotional and behavioral problems was moderated by parental distress. Thus, more research is necessary to draw reliable conclusions.

In the exploratory moderation analyses with positive MM as a predictor, we found a moderation effect of parental distress. Specifically, positive MM predicted less infant regulatory symptoms more strongly under lower levels of both parenting stress and psychological distress compared to higher levels. This further reinforces the notion that MM may serve as a protective factor against children's emotional and behavioral difficulties, particularly under low parenting stress (cf. Hobby et al., 2022, regarding internalizing problems). Surprisingly, when global psychological distress was high, positive MM was associated with more regulatory symptoms. This could imply that positive MM is not inherently adaptive. One explanation is that positive MM in highly distressed mothers is a sign of over‐engagement or intrusiveness. This could be understood as an attempt by highly distressed mothers to engage their children in play. However, over‐stimulating patterns in mother–infant interactions have been related to infant's passivity, irritability, or avoidance, and are considered dysfunctional communication patterns that may evoke or maintain infant behavior (Georg et al., 2018). This again points to the need for further research on the functionality of MM valence indicators, both in relation to effects on later child developmental outcomes and in the context of other interaction parameters and maternal psychological distress.

### 4.5 Limitations

When interpreting our results, some limitations need to be considered. Firstly, we based our hypothesis on the theoretical assumption that parents' MM exerts influence on their infant's regulatory symptoms, which is supported by some empirical evidence from longitudinal studies regarding child emotional and behavioral problems (e.g., Colonnesi et al., 2019). However, the cross‐sectional design of our study does not allow causal interpretations. Therefore, as already discussed, we encourage replications of our findings in longitudinal and cross‐lagged studies. Secondly, the inter‐rater agreement was poor for positive MM on the level of (raw) frequency data. However, the ICC for the dichotomized variable that we created for statistical reasons showed a sufficient (moderate) agreement, which is why we assume the data reliably reflect the valence construct. However, this adjustment resulted in a low resolution in our data, which must be considered in the interpretation. Thirdly, the distributions of the MM indicators were right‐skewed, and especially negative MM was rarely observed. We accounted for this problem by using non‐parametrical tests, dichotomizing positive MM, and excluding negative MM from the hypotheses and analyses. Other studies found low frequencies of negative MM as well and therefore excluded the variable from further analyses (e.g., Demers et al., 2010b; Hobby et al., 2022; McMahon & Meins, 2012). Nevertheless, there is limited evidence (Larkin et al., 2021; Walker et al., 2012) that negative MM is associated with clinical child variables and more research is needed to find out whether this can be replicated. In this vein, additional contextual variables should be explored that could highlight influential factors for negative MM, for example, the investigation of negative MM in the context of acute stress and outside the laboratory. Further, an extension of the coding manual could be helpful to better detect negative MM. In this regard a conceptual refinement of the valence construct, e.g., through a more systematic consideration of the tone of voice, could be beneficial. A conceptual revision could also potentially increase inter‐rater agreement on the valence indicators. Fourthly, the sample predominantly comprised mothers with German background and relatively high socioeconomic status. Furthermore, mothers' high psychological distress levels in our study could partly reflect disturbed sleep in the context of child night‐waking disorders. Hence, it is not possible to generalize our findings to other samples of mothers with higher psychological distress levels other than in the context of infant regulatory disorders or to fathers or more socioeconomically diverse samples. Fifthly, the CompGrp was small, limiting the power of statistical comparisons between the ClinGrp and CompGrp. Therefore, the group comparisons might not reflect the true differences. Future studies should, for example, investigate whether the strength of the associations between MM indicators and parental distress differs in clinical versus comparison groups. Lastly, MM was assessed during free‐play interactions without an additional stress induction, and parental distress was measured using questionnaires regarding broad stress‐ and psychopathology‐related constructs. Future studies that aim to gain a deeper understanding of the dynamics between MM and parental distress should observe the variables in daily life and more stressful contexts and include physiological measures of parental distress.

### 4.6 Clinical implications

Understanding the challenges of taking a mind‐minded stance towards children with infant regulatory symptoms or disorders could be important to guide the support of families (e.g., parent‐focused emotional support and interventions aiming at increasing MM). Based on our results, one approach for early childhood facilitators working with infants with regulatory symptoms could be reinforcing appropriate and positive MM in parents. Particularly in clinical samples of infants with regulatory disorders, it could be beneficial to encourage parents to positively acknowledge their children's mental states. This could be achieved, for example, by providing psychoeducational information on child development to provide context and meaning regarding child regulatory difficulties.

## CONFLICT OF INTEREST STATEMENT

The authors declare no conflicts of interest.

## Supporting information



Supporting Information

## Data Availability

The data that support the findings of this study are available on request from the corresponding author, AKG. The data are not publicly available due to them containing sensitive information.
