# Supplementary material for: Holding dysregulation in mind: How maternal mind‐mindedness relates to regulatory symptoms and disorders in infancy
Source: Infant Ment Health J. 2025 May 18;46(6):855–69. doi: 10.1002/imhj.70020 (PMC12644299; doi:10.1002/imhj.70020)
Supplement: Supplementary file 1 — Supporting Information [file IMHJ-46-855-s001.docx]

**Table A**

*Demographic and clinical characteristics in the clinical (N = 124) and excluded group (N = 30) and group comparisons*

| Characteristic | ClinGrp | Exclusions | ClinGrp vs. Exclusions |
| --- | --- | --- | --- |
| Infant |  |  |  |
| Female, *n* (%) | 58 (46.77) | 9 (42.86) | χ²(1) = 2.13 |
| Age, months, *M* (*SD*) | 8.58 (3.16) | 8.55 (2.85) | *U* = 1882 |
| First-born, *n* (%) | 77 (62.60) | 20 (66.67) | χ²(1) = 0.04 |
| QCFS, *M* (*SD*) | 1.88 (0.31) | 1.90 (0.28) | *t*(152) = 0.29 |
| Mother |  |  |  |
| Age, years, *M* (*SD*) | 33.26 (4.49) | 33.25 (4.07) | *t*(152) = 0.01 |
| High school/higher education, *n* (%) | 88 (70.97) | 25 (90.00) | χ²(1) = 3.16^+^ |
| German origin, *n* (%) | 120 (96.77) | 13 (43.33) | χ²(1) = 54.13*** |
| Married, *n* (%) | 96 (76.461) | 25 (80.00) | χ²(1) = 0.21 |
| SCL-GSI, *M* (*SD*) | 0.55 (0.36) | 0.56 (0.43) | *U* = 1841.5 |
| PSI-parent, *M* (*SD*) | 2.93 (0.64) | 2.84 (0.77) | *t*(152) = –0.14 |

*Note*. QCFS = Questionnaire for Crying, Feeding, and Sleeping, subscale for crying, feeding, and sleeping; SCL-GSI = Global Severity Index of the Symptom-Check-List-90R-S; PSI-parent = parent scale of the Parenting Stress Index. ^+^*p* <.10. **p* <.05. ***p* <.01. *** *p* <.001.
